# Mechanisms, Cofactors, and Augmenting Factors Involved in Anaphylaxis

**DOI:** 10.3389/fimmu.2017.01193

**Published:** 2017-09-26

**Authors:** Rosa Muñoz-Cano, Mariona Pascal, Giovanna Araujo, M. J. Goikoetxea, Antonio L. Valero, Cesar Picado, Joan Bartra

**Affiliations:** ^1^Unitat d’Allergia, Servei de Pneumologia, Hospital Clinic, Universitat de Barcelona, Barcelona, Spain; ^2^Institut d’Investigacions Biomediques August Pi i Sunyer (IDIBAPS), Barcelona, Spain; ^3^Servei d’Immunologia, Centre de Diagnòstic Biomèdic (CDB), Hospital Clinic, Universitat de Barcelona, Barcelona, Spain; ^4^Allergy and Immunology Department, Universidad de Navarra, Navarra, Spain

**Keywords:** adenosine, anaphylaxis, cofactor, exercise, IgE, IgG, mast cell, non-steroidal anti-inflammatory drug

## Abstract

Anaphylaxis is an acute and life-threatening systemic reaction. Many triggers have been described, including food, drug, and hymenoptera allergens, which are the most frequently involved. The mechanisms described in anaphylactic reactions are complex and implicate a diversity of pathways. Some of these mechanisms may be key to the development of the anaphylactic reaction, while others may only modify its severity. Although specific IgE, mast cells, and basophils are considered the principal players in anaphylaxis, alternative mechanisms have been proposed in non-IgE anaphylactic reactions. Neutrophils, macrophages, as well as basophils, have been involved, as have IgG-dependent, complement and contact system activation. A range of cationic substances can induce antibody-independent mast cells activation through MRGPRX2 receptor. Cofactors and augmenting factors may explain why, in some patients, food allergen exposure can cause anaphylaxis, while in other clinical scenario it can be tolerated or elicits a mild reaction. With the influence of these factors, food allergic reactions may be induced at lower doses of allergen and/or become more severe. Exercise, alcohol, estrogens, and some drugs such as Non-steroidal anti-inflammatory drugs, angiotensin-converting enzyme inhibitors, β-blockers, and lipid-lowering drugs are the main factors described, though their mechanisms and signaling pathways are poorly understood.

## Introduction

Anaphylaxis is an acute, life-threatening, systemic reaction caused by the mediators released from different cells (1). Although the underlying mechanism is frequently IgE-dependent, some other mechanisms there are also involved (2–4). Complement activation, neuropeptide release, T-cell activation, immune complex formation, cytotoxicity, IgG-dependent activation, induction of purinergic metabolism, and activation of the receptor MRGPRX2, are some of those alternative pathways (2, 5). Mast cells are considered the pivotal cells in IgE-mediated anaphylaxis (6), and the role of macrophages and neutrophils has been described in IgE-independent reactions (6, 7). Interestingly, basophil implication has been suggested in both IgE-dependent and -independent reactions, although its role in allergic reactions has been considered as redundant with mast cells for some time (8). In humans, CD203c and CD63, basophil activation surface markers, are used to confirm allergen sensitization (9). Recently, a decrease on circulating basophil and an increase in serum concentration of the major basophil chemotactic factor (CCL2) during food and hymenoptera-induced anaphylaxis has been observed, suggesting the role of basophil in human IgE-dependent anaphylaxis (10).

## Immune-Mediated Anaphylaxis

### IgE-Mediated Reactions

The more common mechanism involved in an anaphylactic reaction is promoted by an allergen recognized by an allergen-specific IgE bound to the FcεRI receptor on the surface of mast cells and basophils. When the signal is sufficiently powerful, mast cell and basophil activation make progress, releasing mediators (11). Those mediators also lead to the amplification of the allergic reaction through the recruitment and activation of other cells involved in the IgE immunological response (12–14). However, this explanation is too simple to understand what take place in an *in vivo* reaction, and sundry factors could influence allergen-dependent mast cell and basophil activation under specific conditions (15).

### IgG-Mediated Reactions

There are six different Fcgamma receptors (FcγRI, FcγRIIA, FcγRIIB, FcγRIIC, FcγRIIIA, and FcγRIIIB), and all of them bind IgG. Among them, FcγRI is considered a high-affinity receptor (16). Most of these receptors induce cell activation, except for FcγRIIB, which induces an inhibitory signal and has been proposed as a key player in IgG subclass-dependent anaphylaxis in a recent study (17).

Mouse models have been used to demonstrate the relevance of IgG in anaphylaxis. A passive systemic anaphylaxis model has suggested that FcγRIII on cells as macrophages and basophils mediates these reactions (3, 4, 18), and platelet-activating factor (PAF) but not histamine (3, 4) is the main mediator involved.

An IgG-dependent mechanism has been also suggested in human anaphylaxis. PAF, mostly associated with an IgG mechanism, has a key role in human anaphylaxis as several authors have suggested. Vadas et al. (19) found increased circulating PAF levels and decreased PAF acetylhydrolase (PAF-AH) activity in proportion to the severity of the anaphylactic reaction (20). Indeed, the lowest levels of PAF-AH activity were related with a 27 times more risk of severe or fatal anaphylaxis compared to normal activity (19, 21).

Several authors have suggested that both IgG and neutrophils may be involved in human anaphylaxis. Muñoz-Cano et al. (5) studied patients with anaphylaxis induced by lipid transfer proteins (LTP) and mediated by IgE. They found an increase of specific anti-LTP IgG1 and IgG3 levels and increased expression of the three genes coding for FcγRI (CD64), an activating receptor (5). It has been shown that FcγRI mediates mast cell and neutrophil activation *via* IgG (22, 23), by both IgG1 and IgG3 (16) also in humans. Interestingly, Muñoz-Cano et al. (5) found specific IgG and anti-LTP IgE in those patients, suggesting that both IgG and IgE pathways may contribute substantially to anaphylaxis. Rispens et al. (24) also found both specific IgE and IgG1 anti-α-gal in patients with galactose-alpha-1,3-alpha-galactose (α-gal) allergy.

Neutrophils, activated through FcγRIV-IgG2, are proposed to play a major role in a mouse model of anaphylaxis (7). They are important PAF producers, and a differential PAF release has been observed in neutrophil-dependent reactions in mice (7, 25). However, PAF is also observed in IgE-mediated reactions in animal models (11). Muñoz-Cano et al. (5) showed that several markers of neutrophil activation and trafficking were highly expressed in patients with IgE-dependent anaphylaxis allergic to LTP. Moreover, the authors found increased levels of reactive oxygen species/reactive nitrogen species, known as a measure of oxidative outburst, suggesting an enhancement of neutrophilic activity. Francis et al. (26) also found increased neutrophil activation markers (myeloperoxidase and CD62L) during an acute anaphylactic reaction.

In the light of these findings, the paradigm of anaphylaxis mediated only by IgE and mast cell/basophil seems not totally accurate. In the LTP particular case, anaphylaxis may be elicited *via* IgE, IgG, or both, with the involvement of neutrophils and not only of mast cells and basophils, although other allergens may act similarly.

### Complement Activation in Anaphylaxis

Monomeric IgG and IgG immune complexes can bind FcγRI receptors (27, 28) and are key in the novel paradigm in human anaphylaxis (IgG anaphylaxis). Furthermore, the complement system can also be activated by immune complexes, resulting in the generation of anaphylatoxins such as C3a (23, 29). Interestingly, C3a has demonstrated a direct effect on mast cell and also a synergistic effect (twofold increase) with FcγRI receptor activation (23). Therefore, the combination of IgG and C3a activation results in greater mast cell activation or activation under circumstances in which neither of the stimuli would elicit maximal release on its own.

Large amounts of the anaphylatoxin C3a have been found in peanut severe allergic reactions by Khodoun et al. (30), in both mouse and human plasma. However, allergens such as milk and egg white did not have the ability to activate complement in humans (30). Therefore, several factor as patient susceptibility (5), cofactors (31) and characteristics of a particular allergen may determine the severity of an allergic reaction.

Reactions with drugs solubilized in therapeutic liposomes and lipid-based excipients have been related with the activation of complement in the absence of immune complex. It is the case of Cremophor EL, a diluent used in the older preparations of propofol and paclitaxel, which has been found to induce complement activation (32).

Finally, it has been also demonstrated that lypopolysaccharides (LPS) can induce a strong activation of the complement and trigger an anaphylactic reaction in a mouse model (33). Recently, Rodriguez et al. (34) demonstrated the role of LPS as a co-stimulus triggering anaphylaxis in a mouse model; specific Pru p 3-induced anaphylaxis was generated after nasal sensitization to Pru p 3 in combination with LPS.

## Non-Immune-Mediated Anaphylaxis

### Contact System Activation in Anaphylaxis

It has been identified as direct or indirect activation of the blood coagulation pathway in allergic reactions mediated by IgE (35). During acute anaphylaxis, an increase of the heparin levels and an activation of the factor XII-driven contact system has been observed, which results in the production of bradykinin (36). In fact, after the analysis of more than 150 deaths associated to anaphylaxis induced by oversulfated chondroitin sulfate-contaminated heparin, the possible role of heparin as a trigger of bradykinin formation through contact activation was suggested (37, 38). Therefore, targeting its generation may be a promising strategy for treatment of severe allergic reactions, importantly those with hypotension (39).

### New Mast Cell Receptors in Anaphylaxis: MRGPRX2

Mast cells are classically activated by IgE antibodies, although a range of cationic substances, called basic secretagogues, can induce antibody-independent activation. Among those secretagogues, there are peptides with pro-inflammatory effects and several drugs. Recently, Mrgprb2, the ortholog of the human G-protein-coupled receptor MRGPRX2, has been described to mediate this activation in a mouse model. This receptor seems to be the target of many small-molecule drugs involved in non-IgE anaphylactic reactions, such as non-steroidal neuromuscular blocking drugs (tubocurarine, atracurium, or ciprofloxacin). This work identified a chemical motif that is common to several of these molecules and may be linked to some of the observed side effects. In conclusion, MRGPRX2 may be considered a potential therapeutic target to reduce some adverse effects induced by some drugs (40).

## Cofactors and Augmenting Factors in Anaphylaxis

The so-called accompanying factors may explain why an allergen can either be tolerated or trigger a mild reaction or, in the same patients, induce a severe anaphylaxis. In the presence of cofactors, reactions become more severe and/or the amount of allergen eliciting the allergic reaction can be lower. According to published data, the presence of those accompanying factors occurs in up to 30% of episodes of anaphylaxis (31, 41). Niggemann and Beyer (42) postulated three categories of risk factors for anaphylactic reactions: first, the *augmenting factors*, which influence the immunological mechanism, such as physical exercise, acute infections, drugs [non-steroidal anti-inflammatory drugs (NSAIDs), proton pump inhibitors], alcohol, and menstruation; second, *concomitant diseases*, such as asthma, mastocytosis, and cardiovascular disease, which are associated with more severe reactions and/or increased mortality; and third, *cofactors*, which do not have any influence on the immunological mechanism, as psychological factors (e.g., emotional stress) or specific allergens. Nevertheless, the lack of knowledge about the mechanisms underlying these risk factors limits a strict categorization. Therefore, for the purposes of this review, the terms cofactor and augmenting factor are used indistinctively.

### Estrogens

Gender differences have been reported in the incidence of anaphylactic reactions, demonstrating that anaphylaxis is more frequent in women than men (43, 44), but only during the reproductive years, suggesting that sexual hormones might play a role. Additionally, like the episodes of asthma or urticaria associated with the menstrual cycle (45, 46), recurrent episodes of anaphylactic reactions around menstruation have been described, pointing at the estrogens or progesterone as the augmenting factors involved (47).

The susceptibility of women to develop anaphylactic reactions observed in clinical studies was also demonstrated in a murine model (48). Female mice were ovariectomized to eliminate the major source of estrogens and the result was the decrease in the severity of anaphylaxis. Moreover, the implant of subcutaneous estradiol-releasing pellets in the ovariectomized mice resulted in an increase in the severity of anaphylaxis. The mechanism involved was not related with the increase in mast cell degranulation, but with an augmentation of the vascular permeability. A higher production of nitric oxide as a result of major expression of endothelial nitric oxide synthase was in fact the cause (48).

### Exercise

Exercise is involved up to 10% of anaphylactic reactions, being one of the more frequent augmenting factors (49). However, the knowledge of its pathogenic mechanism still continues to be poorly understood, and some theories have being proposed. Although, according to the literature, the foods involved in food-dependent exercise-induced anaphylaxis are very diverse, wheat is the most frequent one, being ω-5 gliadin the culprit protein in most cases (50). As a result, most of the mechanistic studies have been performed in patients with wheat-dependent exercise-induced anaphylaxis (WDEIA).

One of the theories hypothesizes that exercise induces an activation of tissue transglutaminase (tTG), resulting in a formation of large ω-5 gliadin/tTG complexes that would facilitate the ω-5gliadin–IgE binding. Nonetheless, no direct evidence of this phenomenon has been found in patients with WDEIA (51).

A second hypothesis establishes that exercise would induce an increase in the intestinal allergen absorption, and as a consequence, an increase of blood allergen concentration and the likelihood to develop an anaphylactic reaction (52, 53). Some murine models of food allergy have demonstrated how physical exercise increases the absorption of allergen from the gastrointestinal tract due to mucosa injury (54, 55). An increase of the core temperature in the gastrointestinal tract due to intense exercise would result in epithelial cell damage owing to the phosphorylation state of tight junction proteins. In addition, another mechanism involved in the mucosal damage may be related with the deviation of blood flow away from the splanchnic arteries to the working muscle, resulting in an ischemia/reperfusion cycle that causes the epithelial damage (56, 57).

Another hypothesis establishes that exercise, due to a direct effect on mast cells, would modify the threshold dose of allergen in patients with WDEIA. It has been described that physical exercise induces an increment of the plasma osmolarity (58), and this increase results in mast cells activation and release of inflammatory mediators (59). Additionally, a previous *in vitro* study showed that IgE activation and hyperosmolar stimuli at the same time have a synergistic effect on IgE-induced mast cell release (60). However, the increase of plasma osmolarity due to physical exercise is only significant when the exercise is strenuous and, in patients with WDEIA reactions, the intensity of the physical exercise is frequently moderate.

### Lipid-Lowering Drugs (Statins)

Lipid-lowering drugs can be considered as a risk factor in anaphylaxis since some studies posited that low plasma levels of low-density lipoprotein (LDL) may augment the risk of severe or fatal anaphylaxis. These drugs increase plasma concentration of PAF by lowering PAF-AH activity (19, 61). In this way, Perelman et al. (62) demonstrated a significant direct correlation between PAF-AH activity and LDL levels in patients with peanut allergy. Moreover, a significant correlation between PAF plasma levels and the severity of anaphylaxis has also been demonstrated (33).

### Non-Steroidal Anti-inflammatory Drugs

Non-steroidal anti-inflammatory drugs are other well-known augmentation factors in anaphylaxis. They have been reported to be present in up to 22% of cases of food-induced severe anaphylaxis, constituting a risk factor with an odds ratio >11 (63). In the Mediterranean area, NSAIDs are involved up to 58% of cofactor-induced food-related anaphylaxis episodes (64) and in up to 33% of cases of anaphylactic reaction induced by LTP (65). Two hypotheses have been proposed to explain the underlying mechanisms involved in food-dependent NSAID-induced anaphylaxis (FDNIA).

The first hypothesis suggests that the increase of gastrointestinal permeability and allergen absorption may account for the augmentation effect of NSAIDs (53). It is well known that prostaglandins play an important role in gastrointestinal mucosa defense and repair. NSAIDs, through prostaglandin inhibition, leave gastrointestinal tissues more susceptible to the injury caused by gastric acid and bile and with less capacity to retrieve the mucosa function (38). Additionally, NSAIDs induce mitochondrial damage that leads to the malfunction of the intestinal epithelial cells and increase of the intestinal permeability (66, 67).

A second hypothesis suggests that NSAIDs have a direct impact on mast cells and basophils IgE activation, amplifying their activation and degranulation (68, 69). However, the underlying mechanisms involved remain still unknown.

Bartra et al. (70) suggested that the enhancing effect of NSAIDs in food allergic reactions might be related with the cyclooxygenase (COX) pathway. Several authors (71–73) have also shown that this effect is a class effect, therefore COX-dependent. Moreover, it has been demonstrated that selective COX-2 inhibitors (nimesulide and etodolac) (68, 69) did not increase the severity of food allergic reactions. Additionally, prostaglandin E_1_, an important prostanoid derived from the COX pathway (74), has been demonstrated to be protective in patients with FDNIA (75).

Adenosine metabolism and A3 receptor (A3R) have been linked with the underlying mechanisms of some diseases exacerbated by NSAIDs, such as NSAID-dependent urticaria (76) and aspirin-induced asthma (77). Interestingly, it has also been demonstrated that the activation of A3R enhances FcεRI-induced granule release in human mast cells (78–80). Moreover, a study that evaluated the transcriptome of patients with FDNIA showed an overexpression of genes related to adenosine metabolism, particularly A3R gene (5). NSAIDs are able to inhibit oxidative phosphorylation of ATP and promote its hydrolysis which entails the release of adenosine (81, 82); therefore, a link between NSAIDs, adenosine, adenosine receptors, and allergic reaction has been suggested.

### Angiotensin-Converting Enzyme Inhibitors and β-Blockers

Angiotensin-converting enzyme inhibitors (ACE inhibitors) and β-blockers have been described as augmenting factors in anaphylactic reactions according to several studies (63, 83, 84). The odds ratio established for β-blockers was 6.8 and 13 for ACE inhibitors. However, other studies concluded that the risk of develop anaphylaxis related to ACE inhibitors and β-blockers does not exist unless both treatments are combined (21, 85, 86). Recently, mast cells were recognized as targets of ACE inhibitors and β-blockers in a murine model, augmenting their activation through FcεRI (86). In spite of the fact of this important data, further epidemiological and *in vivo* and *in vitro* studies in humans are necessary to determinate the real impact of these drugs as a risk factors in anaphylaxis.

### Alcohol

Alcohol is involved in up to 15% of cases of anaphylactic reaction according to some series (49, 87), independently of their severity (86). Although, the underlying mechanisms are not well established, alcohol may increase allergen absorption. It has been described that alcohol induces a modification in the expression of the tight junction-associated proteins ZO-1 and claudin-1 of the intestinal epithelium, thereby augmenting the permeability of the intestinal epithelial barrier (88).

An adenosine-related mechanism has also been suggested in IgE-mediated anaphylaxis when alcohol is involved. Alcohol inhibits the adenosine uptake, inducing an increase of the extracellular adenosine, thus enhancing FcεRI-induced mast cells and basophil activation (89).

Another mechanism postulated is based on the capacity of alcohol to boost the serum IgE concentration (90). In a murine model, alcohol intake was linked with a raise in IgE serum levels and a decrease in IgG (90). Nevertheless, this acute alcohol intake has also been linked with lower release of mast cell mediators, such tryptase (91).

## Conclusion

**Figure 1:**
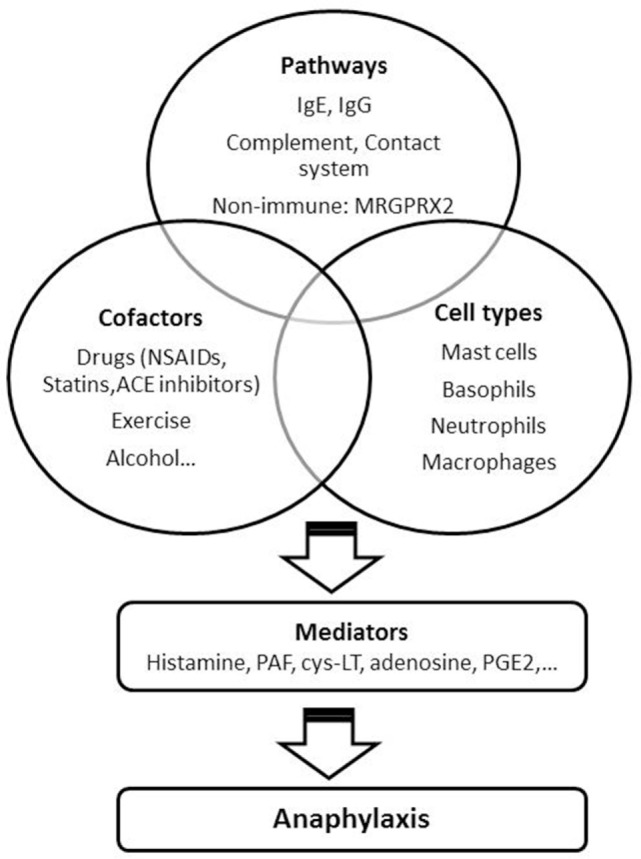
Factors involved in anaphylaxis. NSAIDs, non-steroidal anti-inflammatory drugs; ACE, angiotensin-converting enzyme; PAF, platelet-activating factor; Cys-LT, cysteinyl leukotrienes; PGE2, prostaglandin E2.

IgE, mast cells and basophils have been considered the main key players in human anaphylaxis for a long time, although alternative mechanisms have been suggested. Neutrophils and macrophages, IgG-mediated, complement, and contact system activation are some of them. A range of cationic substances can induce antibody-independent activation through the recently described receptor MRGPRX2. The presence of the so-called cofactors (accompanying or augmenting factors) may explain why the intake of some food sometimes lead to anaphylaxis, while in other cases the same allergen induces a milder reaction or is even tolerated. An understanding of the mechanisms underlying the anaphylactic reactions as well as of the predisposing and augmenting factors could help in the development of new prophylactic and therapeutic approaches. These strategies should target the specific pathways involved in anaphylaxis which, in the light of this review, may be more than one (Figure 1).

## Author Contributions

All authors have contributed equally to this review.

## Conflict of Interest Statement

The authors declare that the research was conducted in the absence of any commercial or financial relationships that could be construed as a potential conflict of interest.
